# Changes of Adipose Tissue Morphology and Composition during Late Pregnancy and Early Lactation in Dairy Cows

**DOI:** 10.1371/journal.pone.0127208

**Published:** 2015-05-15

**Authors:** Ákos Kenéz, Anna Kulcsár, Franziska Kluge, Idir Benbelkacem, Kathrin Hansen, Lena Locher, Ulrich Meyer, Jürgen Rehage, Sven Dänicke, Korinna Huber

**Affiliations:** 1 Department of Physiology, University of Veterinary Medicine, Hannover, Germany; 2 Department of Physiology and Biochemistry, Faculty of Veterinary Science, Szent István University, Budapest, Hungary; 3 Higher National Veterinary School, Algiers, Algeria; 4 Clinic for Ruminants with Ambulatory and Herd Health Services, Center of Veterinary Clinical Medicine, Ludwig Maximilian University, Oberschleissheim, Germany; 5 Institute of Animal Nutrition, Friedrich-Loeffler-Institute Federal Research Institute for Animal Health, Braunschweig, Germany; 6 Clinic for Cattle, University of Veterinary Medicine, Hannover, Germany; Faculty of Biology, SPAIN

## Abstract

Dairy cows mobilize large amounts of body fat during early lactation to overcome negative energy balance which typically arises in this period. As an adaptation process, adipose tissues of cows undergo extensive remodeling during late pregnancy and early lactation. The objective of the present study was to characterize this remodeling to get a better understanding of adaptation processes in adipose tissues, affected by changing metabolic conditions including lipid mobilization and refilling as a function of energy status. This was done by determining adipocyte size in histological sections of subcutaneous and retroperitoneal adipose tissue biopsy samples collected from German Holstein cows at 42 days prepartum, and 1, 21, and 100 days postpartum. Characterization of cell size changes was extended by the analysis of DNA, triacylglycerol, and protein content per gram tissue, and β-actin protein expression in the same samples. In both adipose tissue depots cell size was becoming smaller during the course of the study, suggesting a decrease in cellular triacylglycerol content. Results of DNA, triacylglycerol, and protein content, and β-actin protein expression could only partially explain the observed differences in cell size. The retroperitoneal adipose tissue exhibited a greater extent of time-related differences in cell size, DNA, and protein content, suggesting greater dynamics and metabolic flexibility for this abdominal depot compared to the investigated subcutaneous depot.

## Introduction

Energy metabolism of dairy cows is continuously subjected to adjustments driven by the varying energy status of the lactation cycle. Consequently, this adaptation process substantially affects the metabolism of adipose tissue as the main organ for energy storage [1–3]. Late lactation and the dry period are characterized by the dominance of an anabolic status allowing storage of triacylglycerols (TAG) in the adipose tissues [4,5]. In contrast, early lactation is a period of catabolism associated with an intensive lipid mobilization. The reason for this is a negative energy balance which arises in high-yielding dairy cows due to the mismatch between high energy demand for lactation and decreased feed intake. This results in well-defined physiological and metabolic processes to maintain sufficient energy flow towards life sustaining processes and milk synthesis [4,5]. This cycle of lipid storage and lipid mobilization, which is a typical metabolic phenomenon in dairy cows, necessarily induces extensive adipose tissue remodeling [6,7].

Within the lactation cycle, the early postpartum period is of special importance in terms of adipose tissue metabolism, as this time period is often affected by pathophysiological events [8,9]. Well-known signs of the ongoing lipid mobilization are the increase of the blood non-esterified fatty acids (NEFA) concentration and the decrease of body condition score (BCS), body weight and back fat thickness [10,11]. Furthermore, the decrease of adipose depot weight [12] and the decrease of adipocyte size [13–15] has been reported as indicators of TAG unloading from adipocytes. A strong relationship between adipocyte size and BCS and body weight was demonstrated [16], and associations between the changes in size, volume and number of adipocytes were described [13]. These led to the conclusion that the decrease and increase of adipose mass during the lactation cycle is controlled mainly by changes in the size of the adipocytes [13,16]. Another area of interest concerning adipose tissue metabolism is how differently subcutaneous and abdominal adipose depots contribute to the overall lipid metabolism. In this respect, the retroperitoneal adipose depot, as one of the abdominal depots, was found to undergo a more pronounced weight loss during early lactation [12]. This was accompanied by a more significant decrease in adipocyte size [15], associated with a potentially more intensive lipolysis due to greater activation of hormone-sensitive lipase [17], compared to the subcutaneous depot. Based on these findings, the retroperitoneal adipose depot was discussed to be preferentially mobilized in times of a negative energy balance, compared with the subcutaneous depot [12,15,17].

Studying the changes of indicative components of adipose tissues and relating these to changes of adipocyte size would help further improve our understanding of the dynamic process of adipose tissue remodeling. Therefore, the present study aimed to describe adipocyte size (detected as cell area in histological sections) and composition (DNA, TAG, β-actin and total protein content) of adipose tissues at 4 distinctive time points in the course of the periparturient period: 42 days (d) prepartum, and 1 d, 21 d, and 100 d postpartum. These measurements were conducted on consecutively collected biopsy samples from the same animals, allowing the description of dynamic changes proceeding during the periparturient period within the adipose depots of individual animals. Both subcutaneous (SCAT) and retroperitoneal adipose tissues (RPAT) were studied to gain more information on depot-selective characteristics of adipose tissue cellularity and remodeling. Results of the present study provide a deeper insight to the structural aspects of adipose tissue plasticity in response to the dynamic changes of energy homeostasis throughout the periparturient period in dairy cows.

## Materials and Methods

### Animals, Feeding and Sampling

Twenty German Holstein cows were used for tissue sampling. The study was conducted at the Institute of Animal Nutrition, Federal Research Institute for Animal Health (Friedrich-Loeffler-Institute, Braunschweig, Germany), and was approved by the Lower Saxony State Office for Consumer Protection and Food Safety (LAVES; Oldenburg, Germany) in agreement with the German Animal Welfare Act (permit number: 33.9-42502-04-11/0444). All surgery was performed under local anesthesia, and all efforts were made to avoid suffering of the animals during the study.

Cows were in their second, third, or fourth lactation, and were selected for this study to achieve homogeneity in body weight, BCS, and milk yield of previous lactation, in order to attenuate possible effects of different condition and merit. All animals were kept in a freestall housing system, were clinically healthy, and were dried off 8 weeks before the expected date of parturition. Cows were fed ad libitum and had free access to water. Diets were formulated as a grass-silage and corn-silage based total mixed ration according to the recommendations of the Society of Nutrition Physiology (Frankfurt am Main, Germany) for transition cows. The study period started when cows reached 42 d prepartum and ended at 100 d postpartum. Body condition score of the cows was registered according to the 5-point scale 42 d prepartum and 1 d, 21 d, and 100 d postpartum.

Adipose tissue biopsy samples were collected from all 20 cows from the SCAT and the RPAT depot 42 d before the expected time of parturition (retrospectively 41.8 ± 1.1 d, mean ± SEM) as well as 1 d, 21 d, and 100 d postpartum, according to [17,18]. After preparation of the surgical field and local anesthesia induced with procaine (Procaine 2%; Selectavet Dr. Otto Fischer GmbH, Weyarn-Holzolling, Germany; without epinephrine), samples from adipose tissues were collected under antiseptic conditions. Firstly, a 3–4 cm skin incision was made in the region of the tail head on alternating sides (right and left) to obtain SCAT. Immediately thereafter, a 3–5 cm skin incision was made to collect the RPAT sample, in the angle between the lumbar transversal processes and the iliac bone. Muscles were dissected until reaching the peritoneum, and biopsy samples were taken directly from the adipose depot localized above the peritoneum. Biopsies of RPAT were obtained each time alternating from the left and right flank. Skin incisions were closed with horizontal interrupted mattress suture pattern (Filovet; Wirtschaftsgenossenschaft Deutscher Tierärzte, Garbsen, Germany). After removal, tissue samples were trimmed of connective and vascular tissue and rinsed thoroughly in ice-cold physiological saline solution to reduce blood contamination. About 500 mg of each tissue sample was immediately embedded in Tissue Tek (Sakura Finetek Europe, Alphen aan den Rijn, The Netherlands) and snap frozen for later histological preparation. The rest of the tissues were collected in plastic tubes and snap frozen in liquid nitrogen for composition analysis. All samples were stored at -80°C until further processing.

### Histomorphometric Analysis of Adipose Tissues

Histological sections of SCAT and RPAT samples obtained from 13 out of the 20 cows were prepared using a Leica Jung CM3000 cryostat at -30°C. In case of all 4 time points the samples of the same 13 cows were used. The samples of the remaining 7 cows were not suitable for histomorphometric evaluation due to technical reasons. Tissue samples embedded in Tissue Tek were cut into 10 μm sections. Six non-adjacent sections of each sample were mounted onto glass slides (Superfrost, Gerhard Menzel GmbH, Braunschweig, Germany). Sections were fixated in 4% formaldehyde for 10 min and then hematoxylin-eosin stained. Meyer’s Hemalaun solution (AppliChem GmbH, Darmstadt, Germany) was applied for 5 min, and 0.25% eosin solution (Sigma-Aldrich, St. Louis, MO, USA) was applied for 2 min to the sections. After dehydration in ethanol and clearing in xylol, sections were covered with a cover slip attached with Depex (Serva Electrophoresis GmbH, Heidelberg, Germany). Tissue sections were visualized using an Olympus IX70 microscope and digitally captured using a Leica DFC 290 camera. Exemplary pictures of hematoxylin-eosin stained tissue slides are shown in S1 Fig. For each sample, cell area (in μm^2^) of 164 ± 13 (mean ± SEM) cells was measured in ImageJ 1.48. Cross-sectional area of cell was assessed by histomorphometry and was referred to as ‘cell size’ throughout the manuscript.

### Composition Analysis of Adipose Tissues

To gain additional information on changes of adipose tissue composition in the course of the periparturient period, DNA, TAG, and total protein content, and β-actin protein expression was quantified in the SCAT and RPAT samples of all 20 cows.

#### DNA Content

To isolate DNA from the samples, 100 mg of tissue was homogenized in 1 ml of DNAzol reagent (Invitrogen, Life Technologies GmbH Darmstadt, Germany) using an Eppendorf pestle. Homogenates were incubated at 60°C for 5 min with continuous shaking at 350 rpm and briefly ultrasonicated to ensure complete cell lysis. Centrifugation at 10,000 g for 10 min at 4°C was performed in order to remove insoluble tissue fragments. DNA in the supernatant was precipitated, washed and then resuspended in 8 mM NaOH solution according to the manufacturer’s protocol. Finally, measured DNA concentrations were corrected for wet weight of the tissue sample and results were expressed as μg DNA per g tissue.

#### Triacylglycerol Content

Triacylglycerol content of tissue samples was measured using a colorimetric kit (BioVision Inc, Milpitas, CA, USA). To prepare homogenates, approximately 500 mg tissue was ground in liquid nitrogen. From this tissue powder, a representative aliquot of 50 mg was weighed and mixed with 1 ml 5% Nonidet P40 detergent solution (Fluka Feinchemikalien GmbH, Neu-Ulm, Germany). This mixture was further homogenized with a FastPrep-24 tissue homogenizer (MPI Biomedicals, Santa Ana, CA, USA) and incubated at 99°C for 5 min with continuous shaking at 300 rpm. Afterwards, samples were centrifuged at 10,000 g for 2 min and the sediment was discarded. Incubation and centrifugation were repeated to maximize lipid extraction yield. Triacylglycerol concentration in the lipid extract was measured according to the manufacturer’s protocol with a spectrophotometer. The measurement was based on the enzymatic hydrolysis of TAG and the colorimetric detection of the released glycerol after oxidation. Finally, measured TAG concentrations were corrected for tissue wet weight and results were expressed as mmol TAG per g tissue.

#### Total Protein Content

Total protein concentration was determined based on the Bradford method. To extract proteins, the same tissue powder was used as for the TAG content measurement. One hundred milligram of this powder was homogenized in a lysis buffer [50 mM HEPES (Carl Roth GmbH, Karlsruhe, Germany), 4 mM ethylene glycol-bis(2-aminoethylether)-N,N,N’,N’-tetraacetic acid (Sigma-Aldrich), 10 mM EDTA (Sigma-Aldrich), 0.1% Triton X-100 (Sigma-Aldrich), 100 mM β-glycerol phosphate (Sigma-Aldrich), 15 mM sodium pyrophosphate (Sigma-Aldrich), 5 mM sodium orthovanadate (Sigma-Aldrich), 2.5 mM sodium fluoride (Sigma-Aldrich) and a protease inhibitor cocktail (CompleteMini, Roche Diagnostics GmbH, Mannheim, Germany)] with a FastPrep-24 tissue homogenizer (MPI Biomedicals). Protein extracts were centrifuged at 10,000 g for 5 min at 4°C. Protein concentration was measured in the corresponding fraction by using Bradford reagent (Serva Electrophoresis GmbH) according to the manufacturer’s protocol. Finally, measured protein concentrations were corrected for wet weight of the sample and results were expressed as mg total protein per g tissue.

#### β-Actin Protein Expression

Protein extracts prepared for protein content measurement were used to quantify β-actin expression by Western blotting. Samples were diluted to 0.5 mg total protein per ml in loading buffer [50 mM Tris-HCl (Sigma-Aldrich), 10% glycerol (Sigma-Aldrich), 2% SDS (Serva Electrophoresis GmbH), 0.1% bromophenol blue (Sigma-Aldrich), 2% mercaptoethanol (Sigma-Aldrich); final concentrations] and heated at 95°C for 5 min. Twenty microliter of the diluted samples were separated by SDS-PAGE on 8.1% hand casted gels and transferred to nitrocellulose membranes by using a Trans-Blot Turbo Transfer System (Bio-Rad Laboratories GmbH, München, Germany). Membranes were blocked in a PBS-based solution containing 5% fat-free milk powder (Carl Roth GmbH) and 0.1% Tween 20 (Sigma-Aldrich) for 1 h at room temperature. Blocked membranes were incubated for 1 h at room temperature with a mouse monoclonal anti-β-actin antibody (catalog number A5441, dilution 1:10,000, Sigma-Aldrich). Afterwards, membranes were incubated with a peroxidase conjugate anti-mouse secondary antibody (catalog number A2304, dilution 1:50,000, Sigma-Aldrich) at room temperature for 1 hour. Immunodetection was performed by incubating the membranes with LumiGlo reagent (Cell Signaling Technology, Danvers, MA) and chemiluminescence was detected by a ChemiDoc XRS+ system (Bio-Rad Laboratories GmbH). Exemplary pictures of membranes are shown in S2 Fig. Bands were quantified by densitometry using Image Lab 4.0 software (Bio-Rad Laboratories GmbH). Finally, membranes were Indian ink stained (Pelikan PBS, Peine, Germany) to control equal loading.

### Statistical Analysis

Cell size data were analyzed for relative (%) frequency distribution, with classification of cells as small (under 3,500 μm^2^), medium (3,500–7,500 μm^2^), big (7,500–11,500 μm^2^), or large (over 11,500 μm^2^). The distribution pattern between time points were analyzed both in SCAT and in RPAT by a one-way ANOVA with a Tukey’s multiple comparison test. Cell size and tissue composition data were tested for normal distribution by the Shapiro-Wilk test. The median of the measured cell size values within each sample was registered and assigned to the corresponding sample as a single ‘cell size’ value. Cell size, DNA content, TAG content, total protein content, and β-actin protein expression of SCAT and RPAT samples were analyzed by a two-way ANOVA for factors ‘time related to parturition’ and ‘tissue depot’ with a Tukey’s multiple comparison test. Level of statistical significance was set at P < 0.05. Statistical tests were performed in GraphPad Prism 6.0.

## Results and Discussion

Late pregnancy and early lactation in dairy cows involves extensive changes in adipose tissue metabolism, being under neuroendocrine control and resulting in structural remodeling. Therefore, sampling times were chosen to give a representative overview of the periparturient period: 42 d prepartum was chosen to reflect metabolic status in the dry period with a positive energy balance, at 1 d postpartum lipid mobilization has already begun typically reflected by high plasma NEFA concentrations, at 21 d postpartum lipid mobilization still lasts due to ongoing negative energy balance, and at 100 d postpartum cows usually just have reached positive energy balance again [4,5,19]. In the current study these changes were supported by the BCS of the cows recorded on the sampling days (Table 1).

**Table 1:** Body condition score (BCS) of dairy cows corresponding to biopsy samplings.

**Day** ^**1**^	-42	+1	+21	+100
**BCS** ^**2**^	3.29^a^ ± 0.12	3.09 ± 0.12	2.89^b^ ± 0.11	3.05 ± 0.12

Body condition score was significantly affected by the time related to parturition (P < 0.01, ANOVA with repeated measures).

^1^Related to parturition

^2^Mean ± SEM (n = 20)

^a,b^P = 0.001 (Tukey’s post-test)

### Histomorphometric Analysis

**Fig 1:**
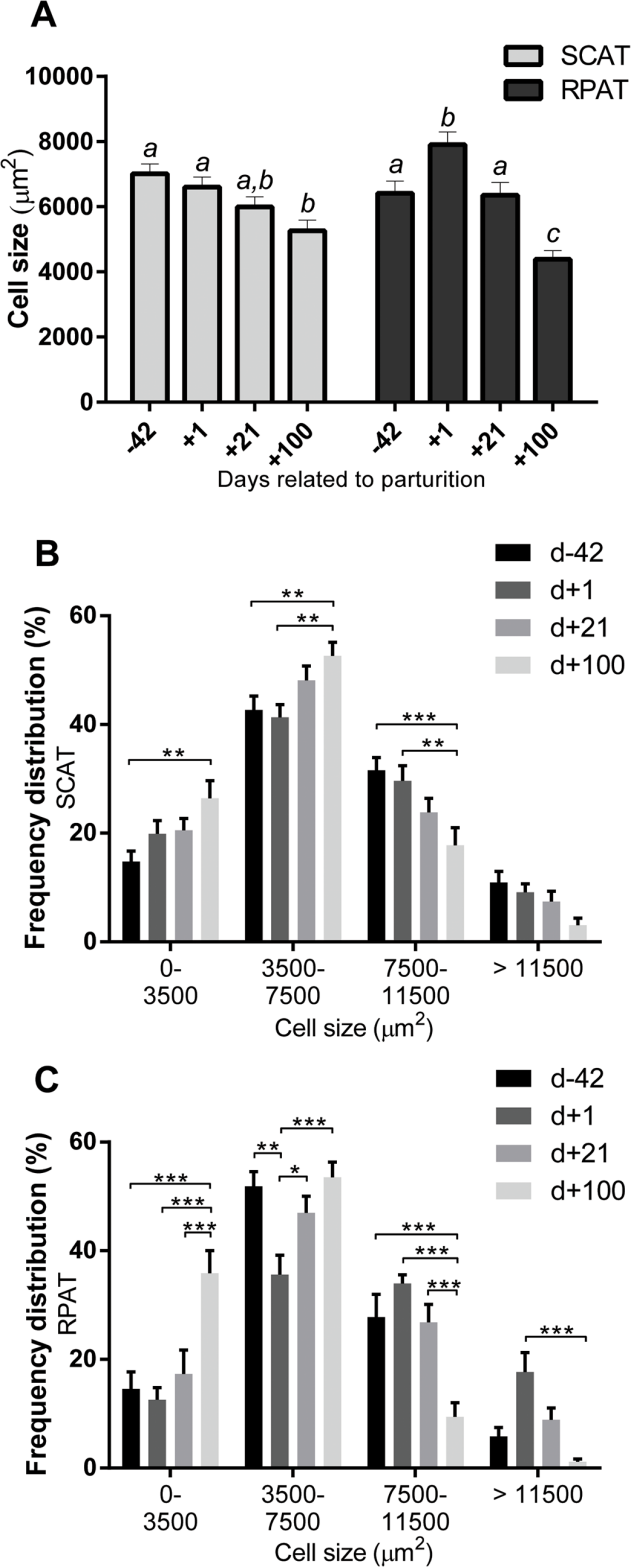
Cell size in subcutaneous (SCAT) and retroperitoneal adipose tissue (RPAT) samples of dairy cows around parturition. **(A)** The median of the measured cell size values was assigned to each sample, and plotted to visualize time-related and depot-related variation. Different superscripts indicate P < 0.05. **(B-C)** Relative frequency distribution of measured cell size values at 42 days prepartum (d-42), and at 1 day (d+1), 21 days (d+21), and 100 days (d+100) postpartum in **(B)** SCAT and in **(C)** RPAT. *P < 0.05, **P < 0.01, ***P < 0.001. Means ± SEM, n = 13.

Adipocytes had a smaller cell size at the end of the trial compared to the beginning (P < 0.001) as shown in Fig 1A. However, the pattern of the differences was not the same in SCAT and RPAT, shown by a significant interaction (P < 0.01) between time course and tissue depot. In fact, SCAT cells were found to become gradually smaller at the 4 studied time points, whereas RPAT cells were the largest at 1 d postpartum, and were thereafter smaller at 21 d and 100 d postpartum (Fig 1A). These time- and tissue-related differences were also reflected in the frequency distribution pattern of the cell size at different time points (Fig 1B for SCAT and Fig 1C for RPAT). Small cells occurred more frequently at 100 d postpartum (P < 0.001 in RPAT), while the highest number of large cells was observed at 42 d prepartum in SCAT and at 1 d postpartum in RPAT (P < 0.001 in RPAT).

The overall decrease in adipose cell size was in agreement with the change from anabolism to catabolism in adipose tissue metabolism of periparturient cows [4,6,7,20,21], and with the findings of Smith and McNamara and Akter et al. [13,15] describing a decrease in adipocyte size during early lactation. The size of adipocytes is mainly determined by their TAG content, and the quantitative changes of the latter are under the control of the net balance of lipogenesis and lipolysis [13,22]. Accordingly, cell size changes shown in Fig 1A indicated a continuous TAG unloading from SCAT cells. In the case of RPAT it was suggested that lipogenesis still dominated in the period between 42 d prepartum and 1 d postpartum, followed by a more intensive TAG unloading in the postpartum period than in SCAT. The assumption that the extent of fat accretion and fat mobilization was greater in RPAT than in SCAT is in fact keeping up with previous work by Locher et al., suggesting greater metabolic flexibility for RPAT [17,23]. Furthermore, the size of adipocytes are known to have a strong relationship with the size of the fat mass stored in adipose depots, as demonstrated by Waltner et al. [16]. In this respect, cell size differences between 42 d prepartum and 1 d postpartum suggested that the RPAT depot gained more weight during the last 6 weeks of pregnancy than the SCAT (see Fig 1A). Subsequently during early lactation, the more pronounced retroperitoneal adipocyte size decrease suggested that the RPAT depot underwent a more intensive weight loss than the SCAT depot. This is consistent with previous work registering a more pronounced weight loss of the RPAT depot than in the SCAT depot in cows during the first 105 d of lactation [12].

The increased number of small cells registered on 100 d postpartum (shown in Fig 1B and 1C) could be explained by an ongoing depletion of the lipid droplet TAG storage, resulting in the ongoing shrinkage of the adipocytes. However, this could also be attributed to preadipocyte differentiation and development of new adipocytes [24–26]. Physiologically, cows are already back to a positive energy balance at 100 d postpartum [1,25,27], which was also indicated in the current study by the increase of BCS at 100 d postpartum (shown in Table 1). The assumption that newly differentiated adipocytes accounted for at least some part of the small cells at 100 d postpartum could also be supported by the findings of Häussler et al. [28], observing a significantly lower percentage of preadipocytes at 100 d postpartum than at 42 d postpartum in bovine RPAT, indicating that many preadipocytes differentiated in this time span.

### Composition Analysis

**Fig 2:**
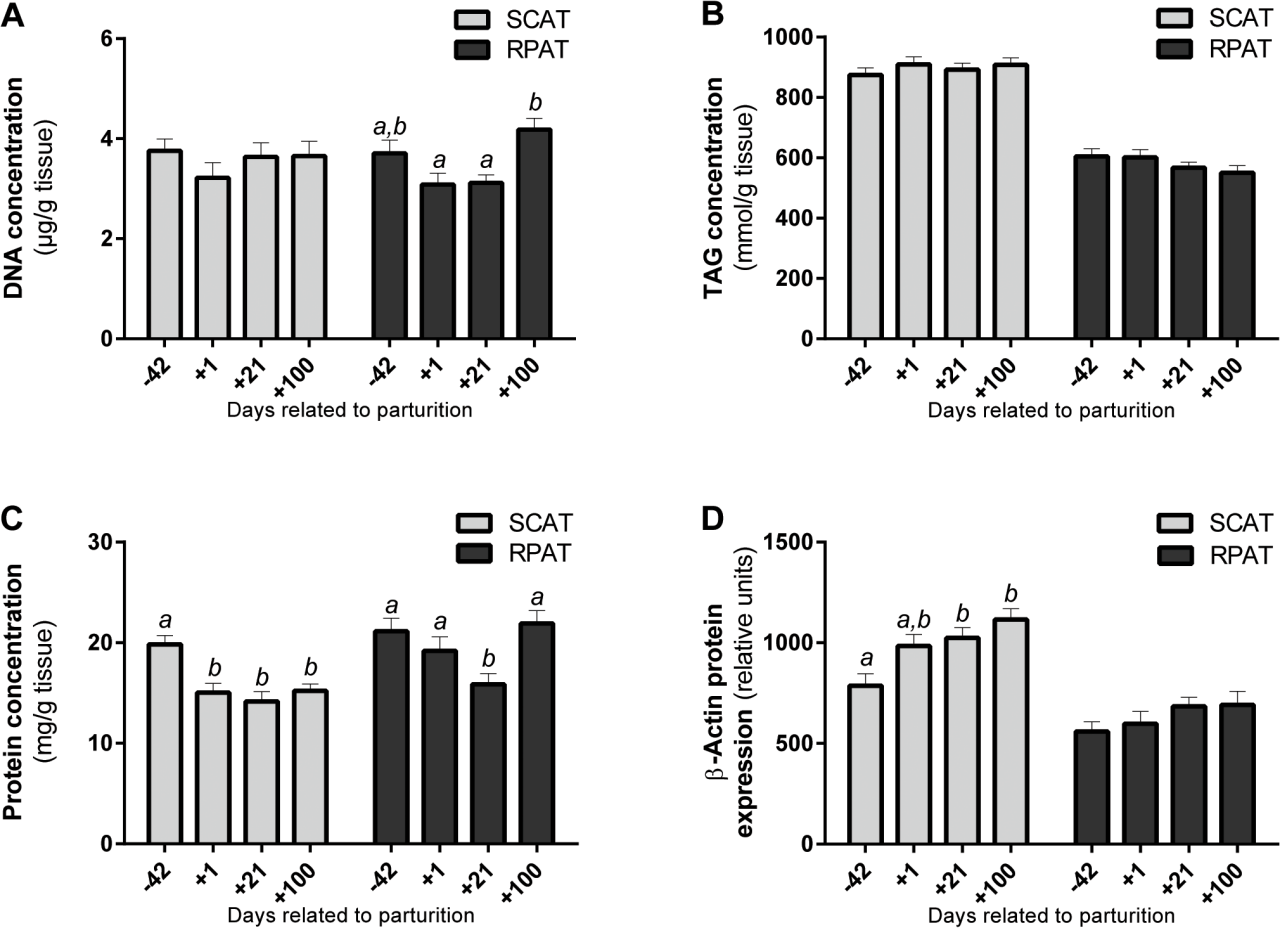
Composition of subcutaneous (SCAT) and retroperitoneal adipose tissue (RPAT) samples of dairy cows around parturition. **(A)** DNA content, **(B)** triacylglycerol (TAG) content, **(C)** total protein content, and **(D)** β-actin protein expression at 42 days prepartum (d-42), and at 1 day (d+1), 21 days (d+21), and 100 days (d+100) postpartum. Different superscripts indicate P < 0.05. Means ± SEM, n = 20.

DNA quantification revealed that the DNA content of RPAT was greater 100 d postpartum than during early lactation (P < 0.05) as shown in Fig 2A. However, SCAT did not have significantly different DNA contents at the observed time points. As the DNA content of a single cell is known to be relatively constant, it was intended to be used as an indicator of cell number [29]. The greater DNA content observed at 100 d postpartum in RPAT suggested that one gram of tissue contained a higher number of cells. This was in accordance with the greater extent of cell size decrease registered postpartum in RPAT, compared with SCAT (Fig 1A).

In contrast to our expectations, TAG content per tissue wet weight was not significantly different between the observed time points (Fig 2B). However, there was a remarkable difference between the depots, as RPAT had a lower TAG content than SCAT (P < 0.001). Despite the ongoing lipid mobilization reflected by gradually smaller cell size values postpartum, TAG content remained stable. Apparently, this was in contrast with previous research revealing that dairy cows can mobilize up to 0.6 kg/day body fat during early lactation [2]. A logical explanation for the lack of change in TAG content might be that TAGs might have had a paramount proportion compared with all other components in adipose tissue. During lipid mobilization TAG was unloaded from the adipocytes, which resulted in a greater number of smaller cells in one gram of tissue, as also demonstrated by Smith and McNamara [13]. In spite of this structural change (i.e. a combination of decrease in cell size and increase in cell density) the overall TAG content related to a weight unit of one gram could still remain nearly unchanged, according to the current results.

Total protein content was lower at all the postpartum time points in SCAT (P < 0.05), and was lower at d 21 postpartum in RPAT compared to the other time points (P < 0.01) as shown in Fig 2C. This was in contrast with the assumption that postpartum mobilization, associated with an unloading of the lipid droplets, necessarily results in a relative increase of the protein fraction of the adipose tissue. The only sign supporting this assumption was the increased protein content observed at 100 d postpartum in RPAT, which again suggested a heavier depletion of lipid stores from RPAT than from SCAT. Furthermore, considering the whole studied period, total protein content was greater in RPAT than in SCAT (P < 0.001).

As shown in Fig 2D, expression of the cytoskeletal protein β-actin was gradually higher at the observed time points throughout the periparturient period in SCAT (P < 0.001). Furthermore, it was significantly lower in RPAT than in SCAT (P < 0.001), in consistence with previous work [23]. The lower proportion of cytoskeletal structure (β-actin) and the higher proportion of total protein (enzymes, receptors, cell organelles etc.) suggested greater metabolic activity for RPAT as an abdominal adipose depot. This was in agreement with Locher et al. [17,23], also concluding an enhanced metabolic flexibility of RPAT based on greater hormone-sensitive lipase and AMP-activated protein kinase phosphorylation rates in this depot in periparturient dairy cows. Furthermore, these findings were in agreement with Akter et al. and von Soosten et al. [12,15] as well, observing a more prominent decrease of RPAT adipocyte size and RPAT depot mass compared to SCAT during the first 105 d of lactation.

The current findings extended previous work investigating adipose tissue of slaughtered cows, by tracking the dynamic changes proceeding during the periparturient period in the same individual animals. However, as a limitation of the study, it has to be noted that the analysis of biopsy samples still provided information of single time points only as snapshots. Consequently, it could not be extrapolated how the studied variables were changing in the time frames between the samplings. Still, studying adipose tissue morphology at these critical time points provided data that allow us to characterize remodeling of SCAT and RPAT in a descriptive manner. Additionally, further mechanistic studies should be conducted addressing the dynamics of metabolic processes that account for the observed differences in adipose tissue morphology.

In conclusion, RPAT was suggested to undergo a more dynamic remodeling process during the periparturient period, implying a higher plasticity of RPAT compared to SCAT.

## Supporting Information

S1 Fig Hematoxylin and eosin stained histological sections of (A-D) subcutaneous adipose tissue and (E-H) retroperitoneal adipose tissue samples of one representative cows at (A,E) 42 days prepartum, and at (B,F) 1 day, (C,G) 21 days, and (D,F) 100 days postpartum. Measure bar indicates 100 μm.
(TIF)

S2 Fig Representative Western blot membranes showing expression of β-actin in (A) subcutaneous adipose tissue, and (B) retroperitoneal adipose tissue samples of dairy cows at 42 day prepartum. (TIF)
