# Supplementary figures and images for: Changes of Adipose Tissue Morphology and Composition during Late Pregnancy and Early Lactation in Dairy Cows
Source: PLoS One. 2015 May 15;10(5):e0127208. doi: 10.1371/journal.pone.0127208 (PMC4433245; doi:10.1371/journal.pone.0127208)

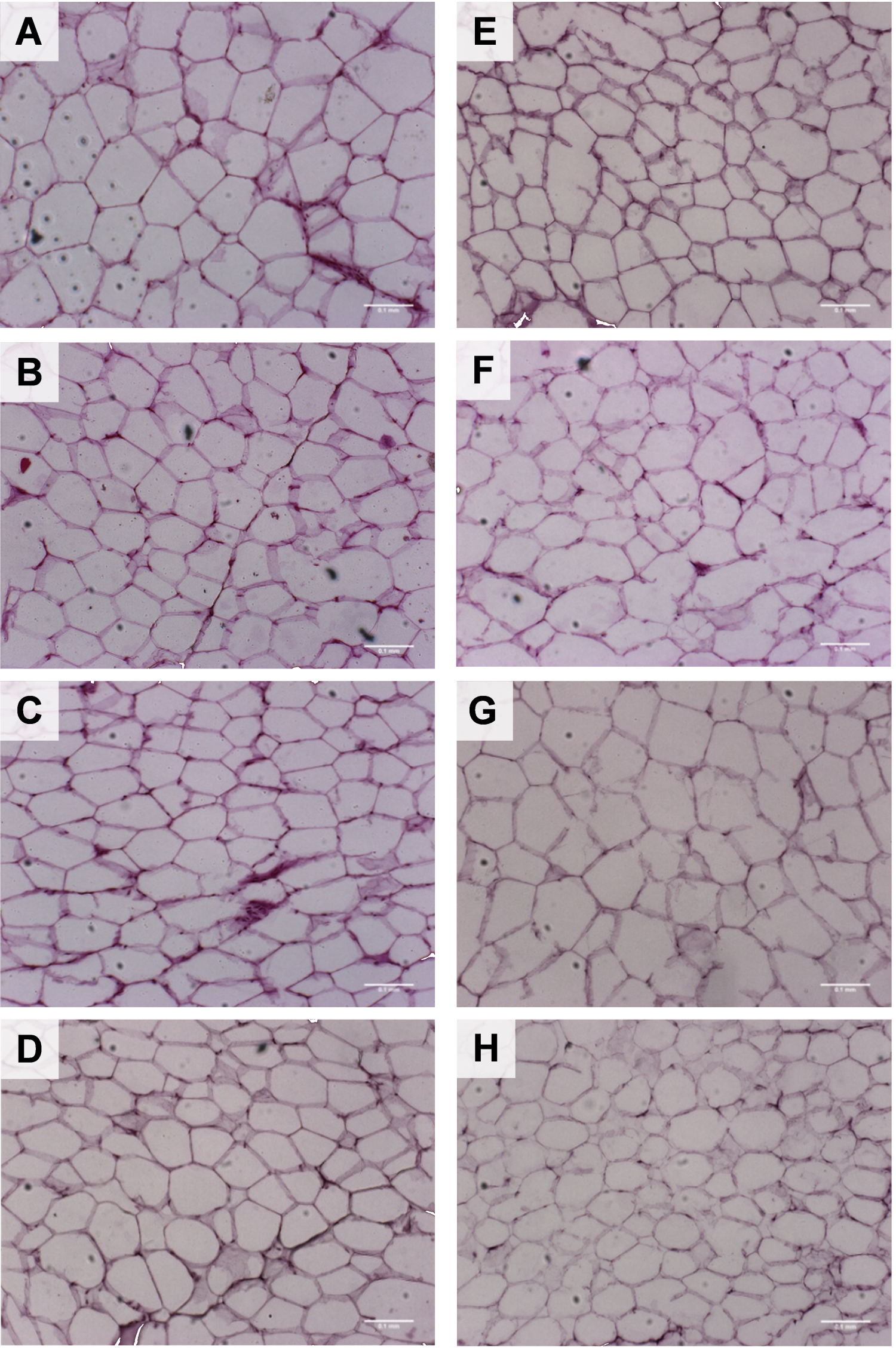

Supplement: S1 Fig — Measure bar indicates 100 μm. (TIF) [file pone.0127208.s001.tif]

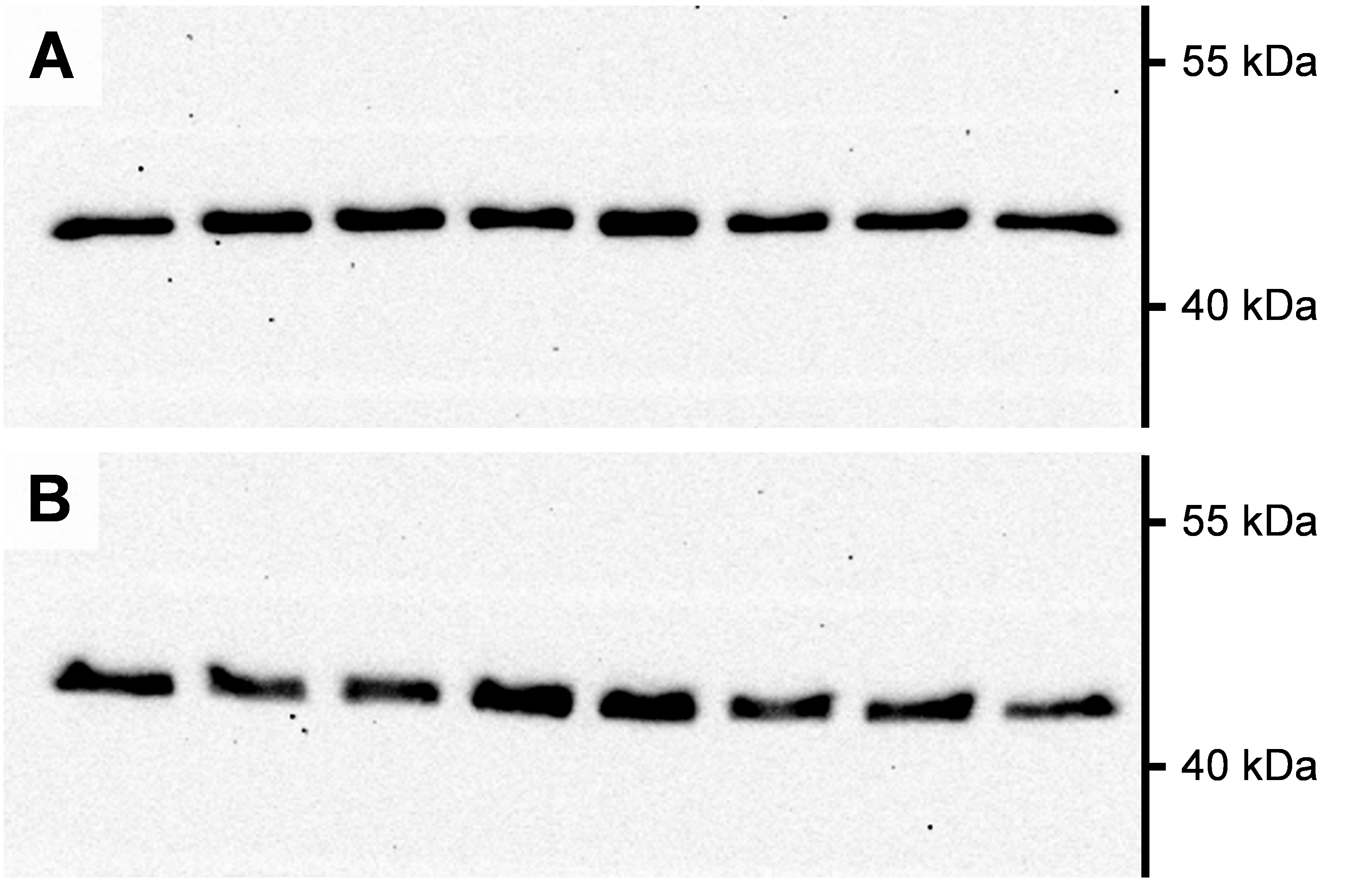

Supplement: S2 Fig — (TIF) [file pone.0127208.s002.tif]
